# The effects of reducing worry in patients with persecutory delusions: study protocol for a randomized controlled trial

**DOI:** 10.1186/1745-6215-13-223

**Published:** 2012-11-21

**Authors:** Daniel Freeman, Graham Dunn, Helen Startup, David Kingdon

**Affiliations:** 1 Department of Psychiatry, Oxford University, Warneford Hospital, Oxford OX3 7JX, UK; 2 Centre for Biostatistics, Institute of Population Health, Manchester University, Jean McFarlane Building, Oxford Road, Manchester, M13 9PL, UK; 3 Academic Department of Psychiatry, Faculty of Medicine, University of Southampton, College Keep, 4-12 Terminus Terrace, Southampton, SO14 3DT, UK

**Keywords:** Delusions, Persecutory, Worry, CBT, Schizophrenia

## Abstract

**Background:**

Our approach to advancing the treatment of psychosis is to focus on key single symptoms and develop interventions that target the mechanisms that maintain them. In our theoretical research we have found worry to be an important factor in the development and maintenance of persecutory delusions. Worry brings implausible ideas to mind, keeps them there, and makes the experience distressing. Therefore the aim of the trial is to test the clinical efficacy of a cognitive-behavioral intervention for worry for patients with persecutory delusions and determine how the worry treatment might reduce delusions.

**Methods/Design:**

An explanatory randomized controlled trial - called the Worry Intervention Trial (WIT) - with 150 patients with persecutory delusions will be carried out. Patients will be randomized to the worry intervention in addition to standard care or to standard care. Randomization will be carried out independently, assessments carried out single-blind, and therapy competence and adherence monitored. The study population will be individuals with persecutory delusions and worry in the context of a schizophrenia spectrum diagnosis. They will not have responded adequately to previous treatment. The intervention is a six-session cognitive-behavioral treatment provided over eight weeks. The control condition will be treatment as usual, which is typically antipsychotic medication and regular appointments. The principal hypotheses are that a worry intervention will reduce levels of worry and that it will also reduce the persecutory delusions. Assessments will be carried out at 0 weeks (baseline), 8 weeks (post treatment) and 24 weeks (follow-up). The statistical analysis strategy will follow the intention-to-treat principle and involve the use of linear mixed models to evaluate and estimate the relevant between- and within-subjects effects (allowing for the possibility of missing data). Both traditional regression and newer instrumental variables analyses will examine mediation. The trial is funded by the UK Medical Research Council (MRC)/NHS National Institute of Health Research (NIHR) Efficacy and Mechanism Evaluation (EME) Programme.

**Discussion:**

This will be the first large randomized controlled trial specifically focused upon persecutory delusions. The project will produce a brief, easily administered intervention that can be readily used in mental health services.

**Trial registration:**

Current Controlled Trials ISRCTN23197625

## Background

Schizophrenia, the core psychotic illness, falls into the top ten medical disorders causing disability worldwide. It contains a heterogeneous collection of symptoms that cluster into many separate factors (for example, [1]). Studying single symptoms has emerged as a way of making progress with the complex problem of schizophrenia spectrum diagnoses. One of the key symptoms is persecutory delusion. This is the unfounded belief that others are deliberately trying to harm the person [2]. In psychosis, persecutory delusions are very frequent (for example, [3]), particularly distressing for patients (for example, [4]), are often acted upon (for example, [5]), and are a predictor of admission to psychiatric hospital (for example, [6]). Paranoid thinking is associated with increased rates of suicide attempts (for example, [7]) and cause particular problems for carers (for example, [8]). Persecutory delusions are a key clinical symptom for which improvements in treatment are greatly needed. Many patients do not respond to neuroleptic medication, relapse is common, and adherence to these treatments is problematic [9]; furthermore, the first generation of generic cognitive behavioral (CBT) approaches only show weak to moderate effects (for example, [10]) and have not been shown to change key causal factors [11]. In the last ten years there have been considerable advances in understanding persecutory delusions but these have not yet been translated into treatment.

### Theoretical rationale

We have developed a theoretical model of the development of persecutory delusions [12,13]. Delusions arise from a number of interacting factors, but worry and associated processing are given a central role in the model. The connection is plausible - worry brings unlikely and distressing ideas to mind and keeps them there - and has been established empirically. It has been shown that worry is extremely common in individuals with persecutory delusions, that it is especially associated with more distressing persecutory delusions, and that it is a predictor of symptom persistence (for example, [14-18]). Other studies have also shown that worry is associated with non-clinical paranoia and predicts its occurrence [19-21]. Furthermore, in a new longitudinal study of over two thousand people taking part in the British Psychiatric Morbidity Survey, worry was shown to predict the new occurrence of paranoid thinking over an 18-month period [22]. Drawing upon the theoretical literature for generalized anxiety disorder, we have shown that worry in individuals with persecutory ideation is associated with catastrophizing (characterized as the worrier posing internal, automatic questions of the form ‘what if this bad thing happens?’) and positive and negative meta-cognitive beliefs [14,16,19].

### The pilot study

On the basis of this work we have completed a pilot study examining the impact of a brief cognitive-behavioral worry intervention for patients with persecutory delusions [23]. The aim was to treat the clinical problem of worry in patients with delusions but also to examine the subsequent impact on persecutory delusions. This is known as an interventionist-causal model approach; ‘it [the interventionist-causal approach] connects causation with the practical interests of psychiatry, defining causation in terms of “what would happen under interventions”, a question of key interest to those of us whose interest is ultimately in intervening to prevent and treat illness’ [24]. Twenty-four patients with persistent persecutory delusions were recruited. Half were randomized to the intervention in addition to their standard psychiatric care and half were randomized to the control group (standard psychiatric care). Assessments were carried out at baseline, end of treatment (one month), and at follow-up (two months). There was a large effect size reduction in worry (Penn State Worry Questionnaire [25]) and also in the persecutory delusions (Psychotic Symptoms Rating Scale [26]). One in three patients showed a 25% or greater reduction in worry and the delusion. Changes in worry were associated with changes in persecutory delusions. However the trial assessments were not carried out blind and the sample size was small. A more rigorous evaluation is now required.

### Research objectives and hypotheses approve

The project has two main objectives:

1.Clinical outcome: To test the clinical efficacy of a brief cognitive-behavioral intervention for worry for patients with persecutory delusions.

2. Explanatory mechanisms: To determine how the worry treatment reduces persecutory delusions.

The trial hypotheses are:

1. A worry intervention will reduce levels of worry in individuals with persecutory delusions.

2. A worry intervention will reduce persecutory delusions, especially levels of distress.

3. The improvements will be maintained at follow-up.

4. The treatment-specific mediator for changes in persecutory delusions will be worry and associated mechanisms (catastrophizing, meta-cognitive beliefs including stop rules, and intolerance of uncertainty).

## Methods/Design

**Figure 1:**
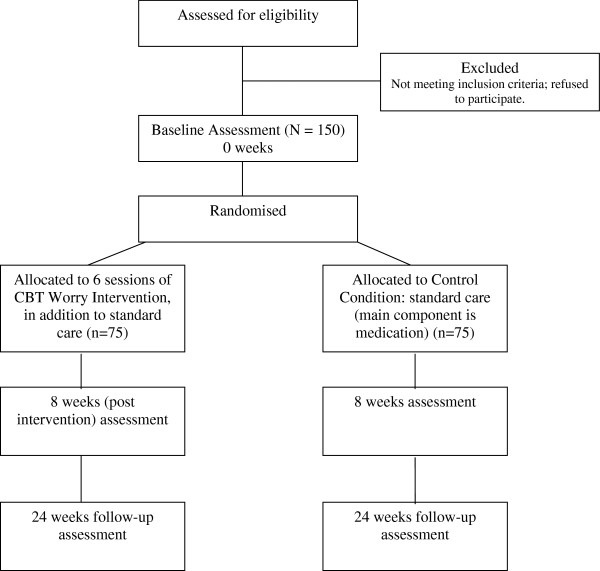
Flow diagram for the Worry Intervention Trial.

The trial is a randomized controlled evaluation. Patients with persecutory delusions will be randomized to the worry intervention in addition to standard psychiatric care or to standard psychiatric care (see Figure 1). A psychological intervention control group is not included in the design. We will instead examine how the treatment works by including repeated measures of worry and associated processes. Non-specific therapist factors will also be assessed [27]. Randomization will be carried out independently, via an on-line system, by the Oxford Cognitive Health & Neuroscience Clinical Trials Unit. Stratification will be by center. Assessments will be carried out by raters blind to allocation. The success of the blinding will be monitored and where there are breaks of blind another assessor will be used. The reliability of the raters on the key interviewer measures will be formally assessed. Embedded within the design will be measures that elucidate how the treatment works. The trial has received a favorable opinion from the NHS Research Ethics Service Oxfordshire REC B (reference: 11/SC/0001). Written informed consent is received from all patients entering the trial. We will follow the MRC Guidelines for Good Clinical Practice in Clinical Trials (1998) [28] in the running of the trial. The Consolidated Standards of Reporting Trials (CONSORT) 2010 Statement, and the extension for non-pharmacologic treatment [29], will be followed for reporting the trial.

### Participants

Patients will be recruited from two mental health National Health Service (NHS) Trusts: Oxford Health NHS Foundation Trust and Southern Health NHS Foundation Trust. Full details of the patient recruitment process are being recorded. The inclusion criteria are as follows: 1) a current persecutory delusion as defined by Freeman and Garety [2]; 2) scoring at least three on the conviction scale of the PSYRATS [26]; 3) that the delusion has persisted for at least three months; 4) a clinical diagnosis of schizophrenia, schizoaffective disorder or delusional disorder (that is, diagnosis of non-affective psychosis (F2) in the International Classification of Diseases and Diagnostic and Statistical Manual IV); 5) a clinically significant level of worry, as indicated by scores above 44 on the Penn State Worry Questionnaire (see [30]); 6) aged between 18 and 65; and 7) where major changes in medication are being made, entry to the study would not occur until at least a month after stabilization of dosage. It should be noted that we will be seeing patients when the main treatment for delusions, neuroleptic medication, has generally been tried at length and their delusions are relatively stable (persistent). Criteria for exclusion are as follows: 1) a primary diagnosis of alcohol dependency, substance dependency, or personality disorder; 2) organic syndrome or learning disability; 3) a command of spoken English inadequate for engaging in therapy; or 4) currently having individual cognitive behavioral therapy (though previous CBT experience is not an exclusion).

### Planned interventions

The worry intervention will be provided in six sessions over eight weeks by two clinical psychologists. This is an increase in the number of sessions used in the pilot, since the patients requested extra sessions. The eight-week window will allow some flexibility for appointment times and the extension of intervals between the final two sessions. The intervention is designed to provide clear and simple messages for patients to take into their day-to-day lives. A series of session booklets have been produced. The worry reduction strategies included are indicated in the anxiety literature to be effective at reducing worry and do not challenge or review the delusion itself. Key influences from the generalized anxiety disorders literature were Butler *et al*. [31], Dugas and Ladouceur [32], Wells [33] and Leahy [34]. The main techniques are psychoeducation about worry, reviewing of positive and negative beliefs about worry, increasing awareness of the initiation of worry and identification of individual triggers, learning to ‘let go’ of worry, use of worry periods, substituting problem-solving in place of worry, and relaxation exercises. Homework exercises are set between sessions. Sessions will be taped for assessment of adherence and for competence [35]. Patients will also be asked to complete an assessment of the therapist’s empathy [36]. Standard care is delivered according to national and local service protocols and guidelines. During hospitalization standard care usually involves prescription of anti-psychotic medication, and to some extent occupational therapy activities and exercise groups. Following discharge, the level of standard care varies according to the needs of the individual. However, this usually consists of prescription of anti-psychotic medication, visits from a community mental health worker and regular outpatient appointments with a psychiatrist. Service use will be measured using the Client Service Receipt Inventory (CSRI) [37]. The CSRI covers services provided by the National Health Service, other health and social care agencies, the criminal justice system and informal carers. Antipsychotic medication data will be extracted from medical records and dosages converted into chlorpromazine equivalents.

### Measures

The key outcome measures will be levels of worry as assessed by the Penn State Worry Questionnaire (PSWQ) [25] and levels of persecutory delusions as assessed by the Psychotic Symptoms Rating Scale - Delusions (PSYRATS) [36]. These are the best available measures of worry and delusions, with established psychometric properties. Secondary outcome measures will be a well-being measure (Warwick-Edinburgh Mental Well-being Scale, WEMWBS) [38], the Paranoid Thoughts Scale (GPTS) [39], the Perseverative Thinking Questionnaire [40], an adapted service user-led outcome measure [41], and the Positive and Negative Symptom Scale [42]. We will also record service use (including medication consumption), adverse events, and hospital admission data using the (Client Service Receipt Inventory; CSRI) [37]. For examination of mediation we will include: the Beck Anxiety Inventory [43], the catastrophizing interview [44,45], the Meta-Cognitions Questionnaire [46], the stop rule checklist [47] and the Intolerance of Uncertainty Questionnaire [48].

At baseline, in order to examine additional moderators of outcome, we will also ask participants to complete assessments of intellectual functioning (Wechsler Adult Intelligence Scale, WAIS) [49], illicit drug use (Maudsley Addiction Profile, MAP) [50], illness and treatment representations [51], probabilistic reasoning [52], and working memory [53].

### Assessment and follow-up

The outcome measures will be completed before therapy (0 weeks), at the end of therapy (8 weeks) and at a follow-up (24 weeks). These can be completed in a single session with a research assessor. The majority of assessments are self-report measures; the interviewer rated PSYRATS and PANSS will be taped for reliability purposes. All the data entry for the two main outcomes will be double checked. The baseline assessment must be completed before randomization. The end of therapy assessment must be carried out after therapy has been completed. We will endeavor to have the repeat assessments carried out at exactly the timings specified, but will allow a two-week window for the post therapy assessment and a one-month window for the follow-up assessment. Participants will be paid £15 for each assessment session, and travel expenses will also be paid.

### Assessment of safety

The following events in trial patients are considered as adverse events: 1. All deaths. 2. Suicide attempts. 3. Serious violent incidents. 4. Admissions to secure units. 5. Formal complaints about therapy. We will also scrutinize any instances of patients being admitted to psychiatric hospital in the period of the therapy. These adverse events are likely to come to the attention of the assessor or therapist but we will also check medical notes at the end of a participant’s time in the trial.

### Sample size

Recruitment will be split equally across centers. In a conservative fashion we power the study to detect moderate effect sizes. In the pilot study the effect sizes were large: worry (PSWQ mean difference = 10.00 SD = 9.50) = 1.05; persecutory delusion (PSYRATS Mean difference = 2.91 SD = 2.15) = 1.35. A simple two-tailed *t*-test with 60 people per group would provide 90% power to detect an effect size of 0.60 at a significance level of .05. It would have 80% power to detect an effect size of 0.52. In practice, further power will be gained by use of multiple regression. Drop-out from the assessments in the pilot was low - 13%. The intervention is brief and the time in the trial will be relatively brief (6 months). Therefore conservatively allowing for 20% drop-out, 150 people will need to be recruited to enable full data to be obtained from 120 participants (60 in each condition).

### Statistical analysis

All main analyses will be carried out at the end of the last follow-up assessments (that is, there will be no interim analyses) and will be based on the intention-to-treat principle, with due consideration being given to potential biases arising from loss to follow-up. Random effects regression models will be fitted to the repeated measures to estimate treatment effects for outcomes, controlling for treatment center, in-patient status and the corresponding baseline assessment for the outcome under investigation. We will allow for the presence of missing outcome data under the assumption that the data are Missing At Random (MAR), using the terminology of Little and Rubin [54], with the possible addition of inverse probability weighting to adjust for the possible role of non-adherence to allocated treatment and other intermediate outcomes as predictors of future loss to follow-up [55]. *Stata* will be used for these main analyses. Secondary analyses to investigate putative meditational mechanisms, but also the effect of receipt of an adequate dose of treatment (CACE estimation), will be carried out; these will use methods similar to those of Baron and Kenny [56] but also the newer approach of instrumental variables analysis to allow for the omitted variables problem that is, hidden confounding [55-58]. *MPlus* and *Stata* will be used for these analyses.

### Research governance

Oxford University is the research sponsor. NHS ethical and R&D approvals have been obtained before trial commencement. For trial management we follow the MRC Guidelines on Good Clinical Practice in Clinical Trials [28]. A Trial Steering Committee (TSC) has been formed, which includes an independent chair and two other independent members, including a service user. A Data Monitoring and Ethics Committee (DMEC) has been formed, which has a clinician as independent chair and a further clinician and statistician.

## Discussion

The first generation of cognitive-behavioral therapies for psychosis now lags behind the transformation in recent years in understanding the causes of psychotic experiences. We have recently discussed how treatments for delusions can be improved [59] by focusing on one putative causal factor at a time, by showing that an intervention can change it, and by examining the subsequent effects on the delusional beliefs. There have now been a number of small pilot studies taking this approach [60,61], but the WIT study will be the first full-scale clinical evaluation. A key clinical advantage for the worry intervention is that rates of engagement seem to be higher because it works on a problem that has been agreed upon with the patient and it does not directly dispute the validity of the delusional beliefs. Furthermore it is a brief, structured intervention, which, if shown to work, could readily be disseminated into mental health services. However it is not suitable for patients with persecutory delusions who do not report worry; this appears, from the empirical literature and our initial recruitment into the trial, to be a small group but full information on this will be gathered for reporting with the trial results. The trial is funded for 30 months and began in October 2011. Final outcome assessments will be complete by the end of November 2013. Therefore the outcome results will become available in 2014.

## Trial status

The trial began patient recruitment in November 2011. Recruitment remains open until July 2013.

## Abbreviations

CBT: Cognitive Behavioral Therapy; DMEC: qData Monitoring and Ethics Committee; EME: Efficacy and Mechanism Evaluation Programme; MAR: Missing At Random; MRC: Medical Research Council; NHS: National Health Service; NIHR: National Institute of Health Research; PSWQ: Penn State Worry Questionnaire; PANSS: Positive and Negative Symptom Scale; PSYRATS: Psychotic Symptoms Rating Scale; TSC: Trial Steering Committee; WEMWBS: Warwick-Edinburgh Mental Well-being Scale; WIT: Worry Intervention Trial.

## Competing interests

The authors’ declare that they have no competing interests.

## Authors’ contributions

DF took the main responsibility for drafting the study protocol. All authors contributed to the design of the trial and read and approved the final manuscript. DF is the main lead of the trial, and takes particular responsibility in Oxford, with DK leading the research in Southampton. GD has the main responsibility for the trial outcome and mediation analyses. HS is the trial coordinator. DF, HS, and DK provide the training and supervision for the trial therapists and research workers.
